# Use of High Throughput Sequencing and Light Microscopy Show Contrasting Results in a Study of Phytoplankton Occurrence in a Freshwater Environment

**DOI:** 10.1371/journal.pone.0106510

**Published:** 2014-08-29

**Authors:** Xi Xiao, Hanne Sogge, Karin Lagesen, Ave Tooming-Klunderud, Kjetill S. Jakobsen, Thomas Rohrlack

**Affiliations:** 1 University of Oslo, Centre for Ecological and Evolutionary Synthesis (CEES), Department of Biosciences, Oslo, Norway; 2 Zhejiang University, Ocean College, Hangzhou, China; 3 Norwegian Sequencing Centre, Department of Medical Genetics, Oslo University Hospital, Oslo, Norway; 4 Norwegian University of Life Sciences, Department of Plant and Environmental Sciences, Ås, Norway; 5 Norwegian Institute for Water Research (NIVA), Oslo, Norway; Laval University, Canada

## Abstract

Assessing phytoplankton diversity is of primary importance for both basic and applied ecological studies. Following the advances in molecular methods, phytoplankton studies are switching from using classical microscopy to high throughput sequencing approaches. However, methodological comparisons of these approaches have rarely been reported. In this study, we compared the two methods, using a unique dataset of multiple water samples taken from a natural freshwater environment. Environmental DNA was extracted from 300 water samples collected weekly during 20 years, followed by high throughput sequencing of amplicons from the 16S and 18S rRNA hypervariable regions. For each water sample, phytoplankton diversity was also estimated using light microscopy. Our study indicates that species compositions detected by light microscopy and 454 high throughput sequencing do not always match. High throughput sequencing detected more rare species and picoplankton than light microscopy, and thus gave a better assessment of phytoplankton diversity. However, when compared to light microscopy, high throughput sequencing of 16S and 18S rRNA amplicons did not adequately identify phytoplankton at the species level. In summary, our study recommends a combined strategy using both morphological and molecular techniques.

## Introduction

Phytoplankton comprises photosynthesizing microscopic organisms that live in almost all fresh and saline water bodies. As the base of the aquatic food web, they are fundamentally important in global atmospheric carbon dioxide acquisition [1]. Assessing the genetic diversity, composition and dynamics of phytoplankton communities is essential to our understanding of how these communities respond to variations in nutrient levels, to invasive species, climate change and other stressors [2]–[5]. Thus, for taxonomical studies focusing on assessing the phytoplankton communities, rapid and precise methods are needed.

Phytoplankton organisms have been visualized and discriminated using light microscopy for over 350 years [6] and light microscopy is still one of the primary techniques in most quantitative studies [7]. However, phytoplankton species are highly diverse with respect to cell size and many are too small to be identified by light microscopy. Since the early seventies, molecular techniques have been developed to detect and discriminate phytoplankton organisms using carbohydrates, toxins, proteins, and nucleic acids as markers [8]–[11]. Among DNA based methods, the high throughput sequencing (HTS) approach has already been successfully applied for the assessment of microbial diversity and micro-planktonic community structure [12]–[14]. However, despite rapid development and wide application of HTS to a broad spectrum of organisms it remains unknown to which extent the results of HTS are consistent with those of traditional approaches. Comparative studies of traditional analysis (i.e. light microscopy) and HTS are therefore needed, but are still rare [8]. In addition, all comparative methodological studies – either in freshwater [15], [16] or coastal systems [14] – are based on a limited number of samples collected during a limited time period (i.e. several seasons). The outcome of such studies may be biased by seasonal variations in the phytoplankton community structure and may therefore underestimate the total phytoplankton diversity. Studies using long time series of samples are therefore needed.

In our current study, phytoplankton composition in a freshwater lake is characterized by light microscopy and HTS, and the results are compared. The eutrophic Lake Gjersjøen, located in southeast Norway, was chosen as the study area. From 1969 to 1989, several projects to control algal biomass were carried out in this lake [17] and the effects on the phytoplankton community were monitored by light microscopy. This resulted in a high-resolution record of phytoplankton composition. In addition, a series of phytoplankton samples, covering the years 1969 to 1989, were taken as filter samples and stored under conditions preserving DNA over longer periods of time. Using this series, the present study determined phytoplankton composition in Lake Gjersjøen using 454 amplicon sequencing of both 16S and 18S rRNA genes (from pooled replicates) and compared these results with those produced by light microscopy.

## Materials and Methods

### Sampling procedures and light microscopy

Lake Gjersjøen is located in the southeast of Norway (59°47′ N, 10°47′ W). The lake has a surface area of 2.6 km^2^, and is a drinking water source for residents in Akershus County. This lake has experienced frequent blooms of toxigenic cyanobacteria that were dominated by *Planktothrix* and *Anabaena*. From 1969 to 1989, water samples were taken by Oppegård waterworks on a weekly basis. Phytoplankton analysis was done shortly after sampling. For phytoplankton analysis, an integrated sample from 0–10 m was taken and a 100 ml subsample was fixed in Lugol’s solution. Of this sample, 2–50 ml were counted using sedimentation chambers according to the method by Utermöhl [18]. For the present study, these phytoplankton data were pooled in one dataset that was used in the analysis. In addition, at the same time of each sampling, 1 L of water was collected from the same water-layers (0–16 m) and filtered through 48 mm cellulose acetate membrane filters with a pore size of 0.8 µm. The samples were air dried, sealed in a plastic bag and kept in darkness at 10–15°C until analyzed.

### DNA extraction

DNA was extracted from 300 filtered and archived water samples representing 9 years of the period 1969 and 1989. Briefly, filters were incubated at 4°C in lysis buffer overnight, and biological materials were transferred from filters into aqueous phase by shaking (3 times 15 seconds at 6800 rpm). The samples were then homogenized by bead beating and incubated in lysozyme for 30 min. After another round of incubation in SDS and Proteinase K (90 min at 60°C), DNA was extracted using the animal tissue kit from Mole Genetics. DNA isolates from the same year were pooled together using Amicon Ultra-0.5 mL centrifugal filters, resulting in nine samples for DNA.

### 16S and 18S tag 454 sequencing

Regions of the bacterial 16S rRNA and eukaryotic 18S rRNA ribosomal small subunit were targeted for amplification and deep-sequencing. For amplification of the 16S rRNA gene, a forward primer (5′ - AGYGGCGIACGGGTGAGTAA - 3′) and a reverse primer (5′ - TCAGCYIACTGCTGCCTCCCGTAG - 3′) were designed to amplify 250 base pairs within the bacterial V2 region. Correspondingly, the V9 region of 18s rRNA was amplified by a forward primer (5′ – CCMGAATTAACTGCCAAAAA–3′) and a reverse primer (5′ – TGATCCTTCTGCAGGTTCACCTAC–3′) with a resulting amplicon of approx. 138 bps. Amplification and 454 sequencing were carried out in two steps according to Sogge et al. [19], but with the given primers for 16S and 18S amplicons. Briefly, in the first step, two parallel polymerase chain reactions were carried out. In the second step, 1.6 µl of PCR product from each parallel PCR reaction were pooled and diluted ten times. A new round of PCR was performed on 1 µl diluted PCR products using the same primers as in round one, but this time GS FLX Titanium Primer A and B were also included in both forward and reverse primers. All forward primers were also ligated with 454 tags to be able to distinguish the samples in downstream analyses. BD Advantage 2 polymerase (BD Biosciences) was used for all polymerase chain reactions. Short PCR products and contaminations were removed using the Sequal Prep Normalization Plate (Applied Biosystems) and Agencourt AMpure XP PCR purification (Beckman Coulter Inc.). Amplicons were sequenced by the 454 FLX Titanium chemistry (454 Life Sciences, Branford, CT) at the Norwegian Sequencing Centre. In total, three pools were sequenced, two 1/8 lanes and one entire Titanium PicoTiter plate. Due to low DNA concentrations, samples for the second 1/8 lane plate were not normalized using the Sequal Prep Normalization Plate (Applied Biosystems).

### Bioinformatics analysis

All sequences were preprocessed using a pipeline outlined in Figure S1. Primer sequences were trimmed off from raw data and low quality sequences were removed according to the assessment of sequencing error rates using QIIME [20]. The precluster command in MOTUR with default settings was used to filter out sequences that most likely contained sequencing errors, by considering their similarities to more abundant sequences. Subsequently, UCHIME was used to identify and remove chimeric sequences [21]. Using MOTHUR [22], identical sequences were grouped and representatively aligned against the SILVA database [23]. The detailed total and unique sequence numbers for each step during 16S and 18S data processing are summarized in Table S2. Finally, 40 862 and 145 035 reads were left in the 16S and 18S datasets, respectively. Using the web-based Bioportal (www.bioportal.uio.no), the remaining high-quality reads were assigned to a taxonomy by blasting against the NCBI nr database [24] for both the 16S and 18S dataset. Subsequently, MEGAN4 [25] was used to display all the species associated with the environmental DNAs. The rarefaction calculations were carried out using the rarefaction analysis command in the software MOTHUR, where we clustered sequences into OTUs by setting a 0.03 distance limit [22]. The HTS sequence sets produced for this study are available under the SRA accession number SRP044824.

### Dataset cleaning and comparison

Dataset comparisons between HTS and light microscopy methods were carried out on two different levels – the species level and genus level. All species (or genera) belonging to the concept of “phytoplankton”, including cyanobacteria (from the 16S sequence set), diatoms, green algae and other kinds of eukaryotic phytoplankton (from the 18S sequence set) were picked out from the cleaned HTS sequence sets. The numbers of OTUs were compared with their corresponding number of phytoplanktonic species (or genera) found by light microscopy. Furthermore, taxa/OTUs detected by both methods were listed as shared species.

### Ethics Statement

No specific permissions were required for field sampling in Lake Gjersjøen. We confirm that the field studies in Lake Gjersjøen (59°47′ N, 10°47′ W) did not involve endangered or protected species. We thank NIVA for providing all the water samples to the Norwegian Sequencing Center (http://www.sequencing.uio.no/) for sequencing and the Bioportal team at the University of Oslo for bioinformatics applications on the Bioportal (www.bioportal.uio.no).

## Results

### Phytoplankton characterized by 16S and 18S rRNA sequencing

**Figure 1:**
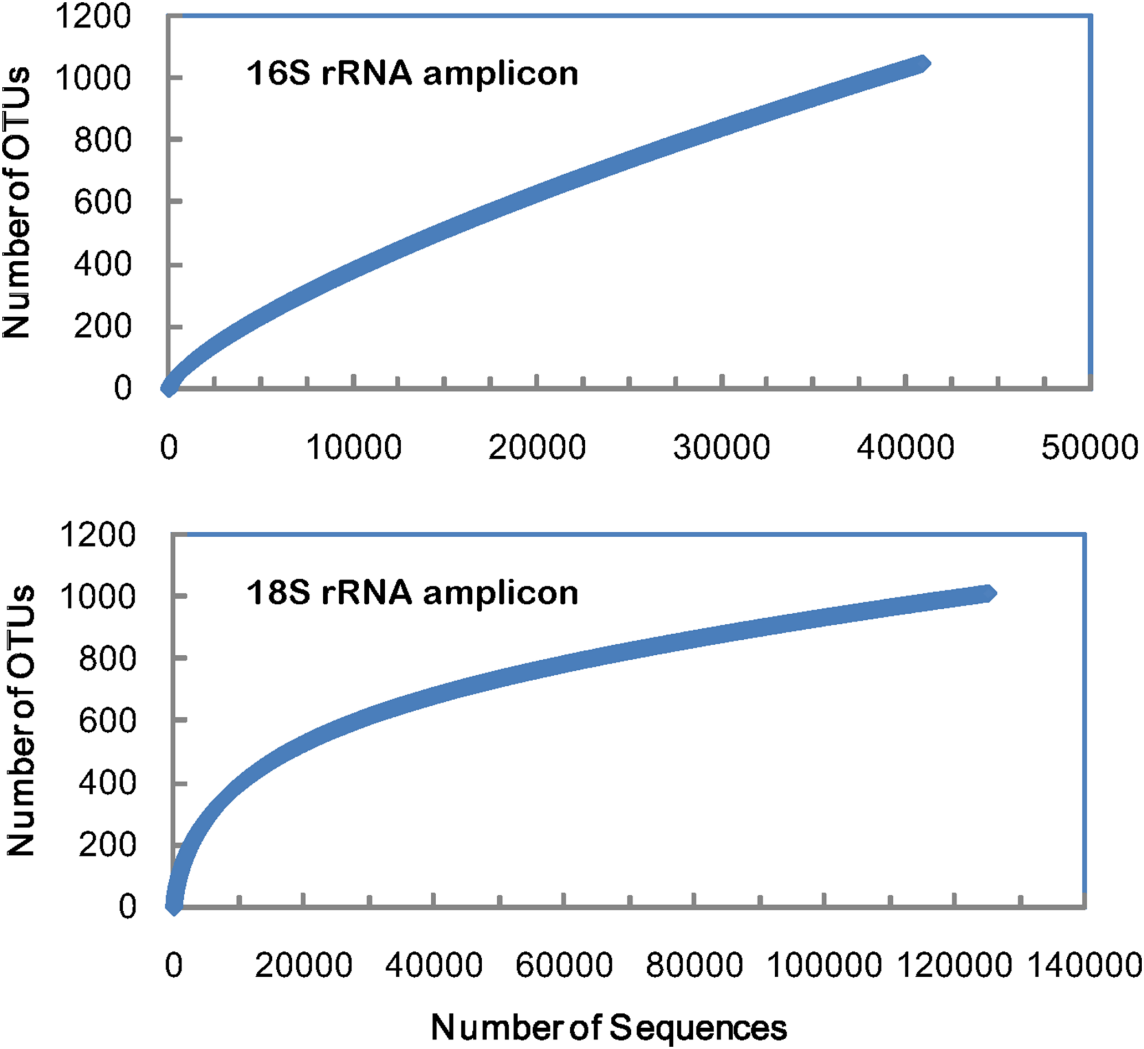
Rarefaction curves of high throughput sequencing of 16S rRNA (V2) and 18S rRNA (V9) hypervariable regions.

After library splitting and sequence denoising of HTS sequence sets, a total of 41,998 and 180,230 reads were generated by PCR followed by 454 amplicon sequencing for 16S and 18S rRNA sequence sets, respectively. Several further quality control steps, which included filtering, preclustering and chimera checking, removed all low quality reads (see Figure S1). A total of 40,862 16S rRNA reads and 145,035 18S rRNA reads remained after quality filtering, which correspond to 1,987 and 2,240 unique sequences (Table S2). Sequence clustering using >97% sequence similarity cut-off resulted in 1,050 16S rRNA OTUs and 1,014 18S rRNA OTUs. Rarefaction curves calculated for both the 16S and 18S sequence sets approached a plateau level (Figure 1), indicating that the reads analyzed for 16S and 18S rRNA hypervariable regions were an accurate representation of the bacterial and eukaryotic diversity in Lake Gjersjøen.

**Figure 2:**
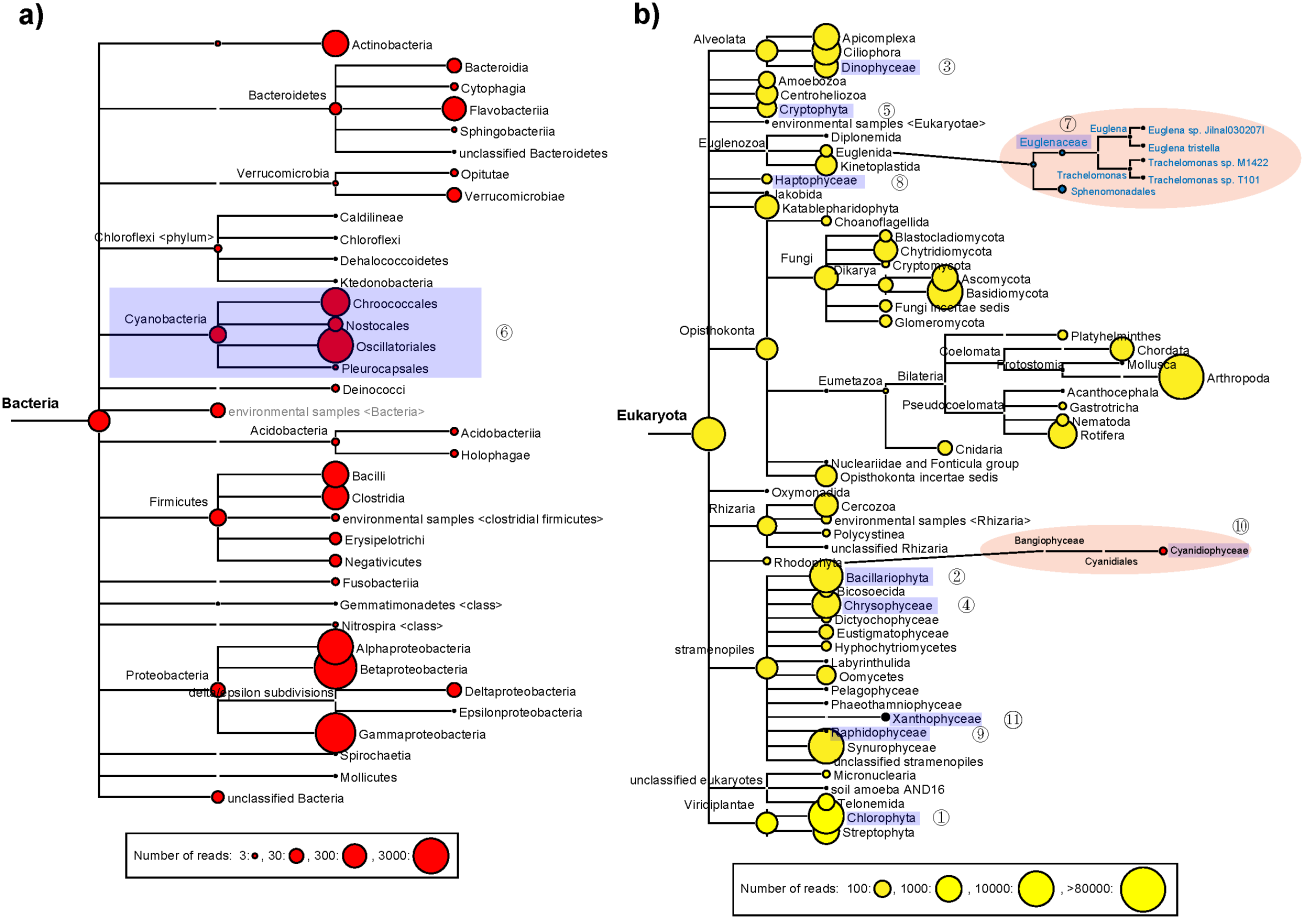
Phytoplankton in Lake Gjersjøen from 1969 to 1989 detected by high throughput sequencing of 16S rRNA (a) and 18S rRNA (b) hypervariable regions.

The 454 amplicon sequencing method revealed a total of eleven classes of phytoplankton in Lake Gjersjøen (Figure 2). The phytoplankton community comprised organisms detected in both 16S and 18S sequence sets, and BLAST matches against NCBI nr databases are shown in Figure 2. Among them, only one class belonged to bacteria - the *Cyanophyceae*, while the other ten classes were all eukaryotic, including *Chlorophyceae*, *Bacillariophyceae*, *Dinophyceae*, *Chrysophyceae*, *Cryptophyceae*, *Euglenophyceae*, *Haptophyceae*, *Raphidiophyceae*, *Cyanidiophyceae*, and *Xanthophyceae* (Figure 2).

Similar to many other freshwater lakes, the *Chlorophyceae*, *Bacillariophyceae*, *Dinophyceae*, *Chrysophyceae* and *Cyanobacteria* were found to be major phytoplankton in Lake Gjersjøen (Figure 2). To make the MEGAN generated schematic phylogenetic trees more concise and readable; we collapsed the 16S phylogenetic tree at class level and phylum level for the 18S phylogenetic tree. The 16S and 18S taxonomic distributions on the species level are also supplied in Figures S2 and S3.

### Comparison of phytoplankton occurrence on species/genus level – high throughput sequencing vs. light microscopy

**Figure 3:**
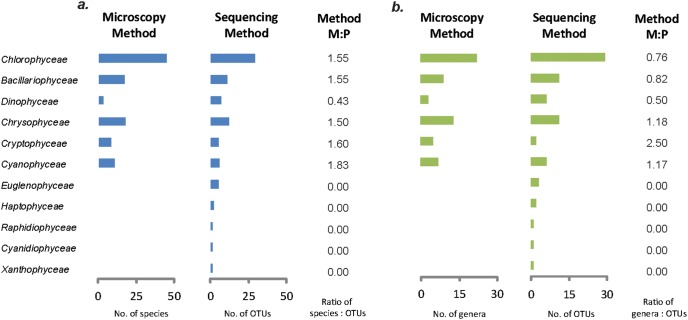
Phytoplankton occurrences in Lake Gjersjøen from 1969 to 1989, and comparison of genera/species numbers using high throughput sequencing of 16S rRNA gene and 18S rRNA gene and microscopy (a. at the species level; b. at the genus level). The M:P stand for the ratio of total species/genus numbers detected by microscopy and HTS.

**Figure 4:**
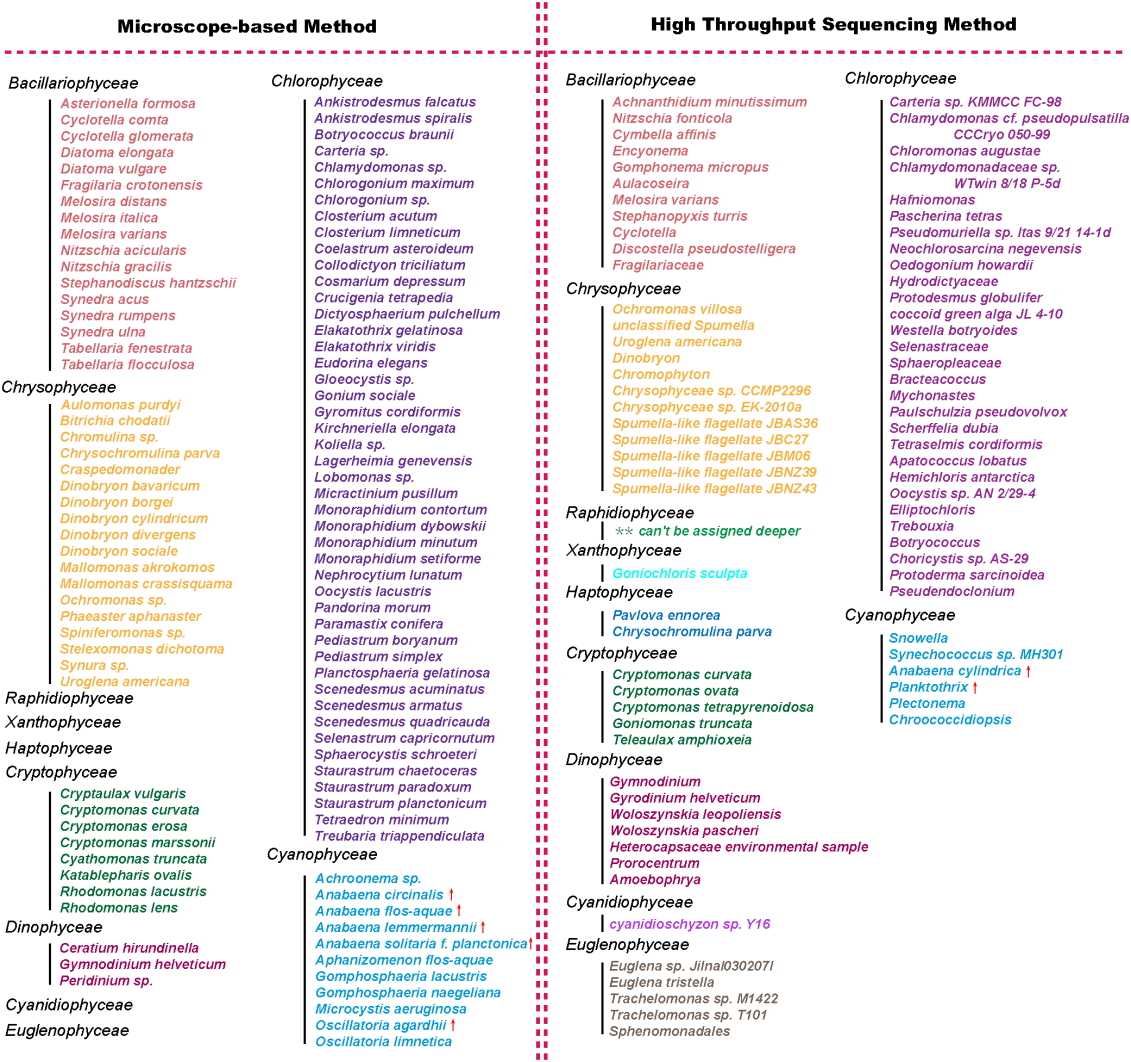
Phytoplankton detected by high throughput sequencing of 16S rRNA gene and 18S rRNA gene and microscopy at the species level in Lake Gjersjøen. The species which formed frequent blooms of toxigenic cyanobacteria in this lake were marked with “↑”.

Patterns of phytoplankton occurrence detected by HTS and traditional light microscopy were compared on species level and subsequently on genus level (Figure 3). Light microscopy detected six phytoplankton classes (totally 58 species) in Lake Gjersjøen from 1969 to 1989 (Figure 3 and 4). In comparison, the HTS method revealed eleven major classes (Figure 3). The undiscovered phytoplankton classes by light microscopy were *Euglenida*, *Haptophyceae*, *Raphidiophyceae*, *Cyanidiophyceae* and *Xanthophyceae* (Figure 3). For the other phytoplankton classes detected by both methods, more different types of species were detected by the traditional light microscopy than the HTS technology. The ratio of total species numbers detected by microscopy and HTS varied from 1.50 to 1.83 for each class, and the only exception is for *Dinophyceae*, where the ratio is only 0.43 (Figure 3a).

Further comparison at genus-level (Figure 3b) fits well with results at species level. The HTS approach exhibited good detection of various phytoplanktonic classes, while traditional light microscopy did not detect as many uncommon or rare phytoplankton classes as HTS (Figure 3b). However, the novel HTS technology could detect higher abundance at genus level than light microscopy. The total number of genera detected by light microscopy vs HTS were 59 and 73, respectively (Figure 3). Furthermore, in comparison to the light microscopy approach, the HTS was also found to be more sensitive in detection of most phytoplanktonic classes (ratio of light microscopy against HTS varied from 0.00 to 0.82), except for *Chrysophyceae* (ratio: 1.18), *Cryptophyceae* (ratio: 2.50) and *Cyanophyceae* (ratio: 1.17) (Figure 3).

### Shared phytoplanktonic species/genus detected by the sequencing and light microscopy approach

As shown in Figure 3, six major phytoplankton classes (from here on and out called “shared” classes) could be detected by both approaches. Species/genus that were detected by both sequencing and microscopy approaches are shown in Table 1. On average, the numbers of OTUs/taxa detected by the two methods were 91 and 66 at the level of species and genus, respectively (Figure 3). Of these, 10 OTUs at species level and 15 different OTUs at genus level were identified as shared OTUs (or taxa) by the two approaches (Table 1). The percentages of shared OTUs at species level (11.0%) and genus level (22.7%) were surprisingly low. Furthermore, the bloom forming cyanobacteria - *Planktothrix* and *Anabaena* were picked up by both methods (Figure 4, Table 1).

**Table 1:** Shared species and genera detected by both high throughput sequencing and light microscopy based on over 300 water samples from 1969 to 1989 in Lake Gjersjøen.

Class	Shared Genus	Shared Species
***Bacillariophyceae***	*Cyclotella*	*–*
	*Melosira*	*Melosira varians*
	*Nitzschia*	*Nitzschia acicularis*
***Chlorophyceae***	*Botryococcus*	*–*
	*Carteria*	*Carteria sp.*
	*Chlamydomonas*	*Chlamydomonas sp.*
	*Oocystis*	*Oocystis sp.*
***Chrysophyceae***	*Dinobryon*	*–*
	*Ochromonas*	*Ochromonas sp.*
	*Uroglena*	*Uroglena americana*
***Cryptophyceae***	*Cryptomonas*	*Cryptomonas curvata*
***Dinophyceae***	*Gymnodinium*	*Gymnodinium helveticum*
***Cyanophyceae***	*Anabaena*	*–*
	*Planktothrix*	*Planktothrix sp.*
	*Snowella*	*–*

## Discussion

In our study, for most classes of phytoplankton, a higher number of species were detected by traditional microscopy than by HTS (Figure 3a and 4), and the opposite was observed at the genus level (Figure 3b). Moreover, the number of taxa detected by both methods was relatively low (Table 1). This discrepancy was somewhat unexpected and requires discussion.

First, the differences in the underlying approaches used for HTS and microscopy methods could result in the observed discrepancies. In the case of the HTS based approach, DNA extraction (e.g. difficulties to break the shell of some phytoplankton like diatoms) and PCR biases (e.g. preferential amplification of some gene variants) have been shown to affect species detection [26]. Further, the databases used for blasting sequences could also cause differences in the species detection. As for the microscopy approach, the results are largely influenced by the expertise of taxonomist. However, in this study all cell countings were accomplished by one researcher, but still, his expertise might have improved over time.

Second, the different approaches applied in microscopy and HTS make the direct comparison of these two methods difficult [14]. Usually, much smaller volumes of water are used for microscopy compared to the volumes filtered for HTS. In the current study, several hundred water samples were pooled together to generate both the microscopy and HTS datasets, which probably eliminates the influences of differences in sampling volumes. However, relatively fresh water samples were analyzed by microscopy, while preserved historical samples were used for the HTS. As demonstrated previously, 10–30% of the freshwater planktonic ciliate cells are lost in fixed water samples after 9 months preservation based on morphological analyses [27]. However, the DNA will still be preserved even the cell is lysed. Although some DNA degradation is expected, the long time series these samples represent in this study is quite valuable.

The limitations of the light microscopy approach may partly provide an explanation for the contrasting results produced by HTS and light microscopy at species level. By the use of HTS the whole composition of ecosystem, including small-sized species, could be detected. With the use of light microscopy such small-sized species may escape detection. An example in our study is *Synechococcus sp.*, one of the most thoroughly investigated pico-algae species [28]. It could be detected by 454 amplicon sequencing but has no records in the microscopy dataset (Figure 4). Traditional microscopy may also overestimate the richness of phytoplankton. Several examples from the literature demonstrate that different phenotypes and transition types of a given phytoplankton species may be identified as separate species [29]. The change in phenotype due to variations in environmental conditions may also cause conspecific individuals to be identified as distinct species [30]. Furthermore, there are cryptic species revealed by molecular studies, existing in many groups. HTS can, in theory, discriminate these cryptic species, which is by definition impossible for optical methods.

Since high throughput sequencing requires little taxonomy pre-knowledge and can produce high throughput data, it has become a powerful tool for phytoplankton identification [8], [31]. However, the taxonomy level applied in HTS studies may seriously affect the results of detections. When higher taxonomic levels are applied, the HTS showed higher accuracy (i.e. at genus-level, the amplicon-sequencing approach was more sensitive) (Figure 3). Similar to our findings, Ovaskainen et al. (2010) found that for the BLAST-based identification of OTUs from wood-inhabiting fungi, higher taxonomic levels are typically identified with higher accuracy than species [32]. In line with these findings, our survey of the HTS sequence set (Figure S4) indicated that the resolution of sequencing data was unable to reliably provide species level identifications, most probably due to short reads generated by 454 HTS. The blast search against the nr database gave many parallel results with similar scores and E-values. For instance, both E-values and scores indicated that the read “Plate2.V9.48_13149” could either be *Asterionella* or *Tabellaria* (Figure S4b). Thus the blast search avoided giving an uncertain taxonomy, and thus *Asterionella*, one of the major diatoms in the microscopy dataset, was absent from our 18S sequencing set (Figure 4). Moreover, it is also possible that some sequences in our 16S and 18S datasets were not yet present in the BLAST-nr dataset. Eiler et al. also found that detailed HTS and microscopy taxa had only low taxonomic correspondence in unveiling distribution patterns of freshwater phytoplankton [16], and the current discrepancies in taxonomic frameworks was thought to be responsible for such disagreement between both methods [16]. Due to the lack of information in the nr dataset and the relatively short reads (approx. 250 bps for 16S amplicon and 138 bps for 18S), the resolution of 16S and 18S sequencing datasets were limited even when the most careful bioinformatic analyses were performed. On the other hand, using too coarse taxonomy may lead to decreasing sensitivity of assessments - and may thus hamper the detection of ecological effects [31]. For example, the difference between natural streams and managed watersheds could not be detected at the family level as at genus/species level [33]. Regarding the trade-off between sensitivity and precision, our results suggest that it is most appropriate to use HTS at genus level when analyzing phytoplankton communities.

HTS has undoubtedly broadened our understanding of microplankton diversity in both freshwater and oceanic ecosystems [4], [5], [15], [31], [34]. Currently the microbial communities are thought to be composed of a low number of high-abundance taxa and a relatively high number of low-abundance taxa [35], [36]. A “rare biosphere”, which refers to low-abundance high-diversity taxa, is also indicated by the HTS approach in our study. These rare species were not detected in the light microscopy dataset (Figure 3). Although low in number of reads (Figure 2), rare species might have an important role within the phytoplankton community [37]. Therefore, to fully assess the diversity of phytoplankton, HTS is certainly a better approach. Considering the limits of using short reads of hypervariable regions for the identification of organisms to lower taxonomic levels, the recent advances in sequencing are probably the solution for the future. The PacBio technology is able to generate long reads with a high consensus accuracy (99.99%) and the paired end Illumina sequencing currently provides 500 bp read lengths (and lengths will increase further) [38]. Hence, it is likely that a better resolution would be achieved by sequencing longer or full length ssu amplicons by the use of additional genes such as LSU, ITSrRNA and/or tufA in combination with ssu.

In summary, this study shows that light microscopy and HTS each have their own strengths and weaknesses. Any DNA based method including HTS will avoid bias due to different levels of taxonomic expertise. It may have a higher resolution and may discriminate between cryptic species. It is also easily adapted to work at different taxonomic levels. In addition, there is no lower size limit as it always will be with optical methods. The advantage of light microscopy is that it has a much lower technology threshold. Therefore a combination of both methods would be best for future phytoplankton research, by which we could capture quantitative changes as well as the total diversity of phytoplankton. Although the accuracy of light-microcopy results depends on taxonomy pre-knowledge that the observer holds and may be biased by the technical skill along with the high cost of specialized training and sample processing time, light microscopy is still one of the primary techniques in phytoplankton research [7]. It requires only relatively cheap equipment, and offers direct description of phytoplankton, which cannot be replaced by DNA-based techniques. However, an integrative approach of both morphological and molecular methods has rarely been employed [14], [39], but as demonstrated here may provide deeper insights into the structure of phytoplankton communities.

## Supporting Information

Figure S1 **Pipeline of the processing of 16S rRNA gene and 18S rRNA gene reads using different bioinformatics software.** As described in the “Methods and Materials” part, several different bioinformatics software including QIIME, MOTHUR and MEGAN were used in the quality filtering steps and phylogenetic analysis. Detailed commands used in the various steps were arranged according to the proceeding order. The input and output file names in each step of data processing are also given in this figure.
(DOC)

Figure S2 **Overview of the 16S rRNA gene sequence set displayed by MEGAN.** The species detected by the 454 high throughput sequencing of 16S rRNA high variable regions were displayed as a schematic phylogenetic tree using the software MEGAN.
(DOC)

Figure S3 **Overview of the 18S rRNA gene sequence set displayed by MEGAN.** All the high quality reads generated by the 454 high throughput sequencing of 18S rRNA complicons were assigned to a taxonomy and displayed as a schematic phylogenetic tree using the software MEGAN.
(DOC)

Figure S4 **The community structure of diatoms in our 18S rRNA gene sequence set (a) and the BLAST output against the nr database for reads assigned to “** ***Fragilariaceae*** **” (b) which gave many parallel results with the same scores and E-value.** It is possible that one representative sequence has two or more taxonomic best matches with an equal BLAST matching score. In that case this OTU could not get its final taxonomy assigned at a level below genus.
(DOC)

Table S1 **Sample descriptions of all the 300 filters used in sequencing approach.** As described in the “Methods and Materials”, in total 300 different stored filters were used in our experiment, environmental DNAs extracted from these filters were pooled together as the DNA templates in the polymerase chain reactions before sequencing. The sampling date, year and sampling depth are all shown in this table.
(DOC)

Table S2 **Number of total and unique sequences during the 16S rRNA gene and 18S rRNA gene sequence processing.** Several different steps such as denoising and chimera checking were carried out during sequence processing (see also Figure S1), low quality reads were filtered out by bioinformatics treatment, all the numbers of remaining sequences (and corresponding unique sequences) were recorded.
(DOC)

Table S3 **Part of microscopy dataset.** More than 5000 records were listed in the whole microscopy dataset. As shown in this sample table, each record includes the following information: (1) sampling date; (2) sampling depth; (3) species code (which could be translated to species name using a code - taxonomy table); (4) phytoplankton class; (5) bio-volume.
(DOC)
