# Supplementary material for: Use of High Throughput Sequencing and Light Microscopy Show Contrasting Results in a Study of Phytoplankton Occurrence in a Freshwater Environment
Source: PLoS One. 2014 Aug 29;9(8):e106510. doi: 10.1371/journal.pone.0106510 (PMC4149573; doi:10.1371/journal.pone.0106510)
Supplement: Figure S1 — Pipeline of the processing of 16S rRNA gene and 18S rRNA gene reads using different bioinformatics software. As described in the “Methods and Materials” part, several different bioinformatics software including QIIME, MOTHUR and MEGAN were used in the quality filtering steps and phylogenetic analysis. Detailed commands used in the various steps were arranged according to the proceeding order. The input and output file names in each step of data processing are also given in this figure. (DOC) [file pone.0106510.s001.doc]

**Figure S1. Pipeline of the processing of 16S rRNA gene and 18S rRNA gene reads using different bioinformatics software.** As described in the “Methods and Materials” part, several different bioinformatics software including QIIME, MOTHUR and MEGAN were used in the quality filtering steps and phylogenetic analysis. Detailed commands used in the various steps were arranged according to the proceeding order. The input and output file names in each step of data processing are also given in this figure.

**Figure S1.**

503_default.fna

**QIIME:** split_libraries.py -m MetaData/metadatfil_plate2_P_V2_60_primer -f Data/split/503_default.fna -q Data/split/503_default.qual -o Analyses/Split_Output/Split_16s -r -b 10 -l 100 -s 20 -z truncate_only -M 2

503_default.qual

**QIIME:** denoiser.py -i Data/split/503_default.sff.txt -f Analyses/Split_Output/Split_16s/seqs.fna -v -o Analyses/Denoiser_Output/Denoiser_16s --primer AGYGGCGNACGGGTGAGTAA -e /site/VERSIONS/Denoiser-0.91/Data/Titanium_error_profile.dat

16s.fasta

**QIIME:** inflate_denoiser_output.py -c Analyses/Denoiser_Output/Denoiser_16s/centroids.fasta -s Analyses/Denoiser_Output/Denoiser_16s/singletons.fasta -f Analyses/Split_Output/Split_16s/seqs.fna -d Analyses/Denoiser_Output/Denoiser_16s/denoiser_mapping.txt -o Analyses/Denoiser_Output/Denoiser_16s/denoised_seqs.fna

denoised_seqs_16s.fna

**mothur** >unique.seqs(fasta= denoised_seqs_16s.fna)

denoised_seqs_16s.unique.fasta

& denoised_seqs_16s.names

**mothur** > align.seqs(fasta=denoised_seqs_16s.fna, reference=silva.bacteria.fasta, processors=2)

denoised_seqs_16s.unique.align

**mothur** >screen.seqs(fasta=denoised_seqs_16s.unique.align, name=denoised_seqs_16s.names, start=2049, end=6436, processors=2)

**mothur** >filter.seqs(fasta=16s.trim.unique.align, vertical=T)

denoised_seqs_16s.filter

**mothur** > pre.cluster(fasta=denoised_seqs.unique.good.filter.fasta, name=denoised_seqs.good.names, diffs=2)

**mothur** >chimera.uchime(fasta=denoised_seqs.unique.good.filter.precluster.fasta, reference=silva.gold.filter.fasta, processors=2)

**mothur** >remove.seqs(accnos=denoised_seqs.unique.good.filter.precluster.uchime.accnos, fasta=denoised_seqs.unique.good.filter.precluster.fasta, name=denoised_seqs.unique.good.filter.precluster.names)

&denoised_seqs_16s.good.unique.filter.unique.fasta

Pre cluster& Chimera checking with Uchime

**mothur** >dist.seqs(fasta=16s.final.fasta,

cutoff=0.25, processors=2)

16s.trim.unique.filter.dist

**mothur** >cluster(column=16s.final.dist,

name=16s.final.names)

16s.final.an.sabund & 16s.final.an.rabund &16s.final.an.list

**mothur** >rarefaction.single(list=16s.final.an.list, label=unique-0.03-0.04-0.05-0.07-0.10, freq=10)

Rarefaction curves based on different similarities

**mothur** >catchall()

**mothur** >summary.single(calc=nseqs-coverage-sobs-npshannon)

Taxonomic analysis in **MEGAN**

Statistical analysis in **R**
