# Supplementary material for: Use of High Throughput Sequencing and Light Microscopy Show Contrasting Results in a Study of Phytoplankton Occurrence in a Freshwater Environment
Source: PLoS One. 2014 Aug 29;9(8):e106510. doi: 10.1371/journal.pone.0106510 (PMC4149573; doi:10.1371/journal.pone.0106510)

**Figure S2. Overview of the 16S rRNA gene sequence set displayed by MEGAN.** The species detected by the 454 high throughput sequencing of 16S rRNA high variable regions were displayed as a schematic phylogenetic tree using the software MEGAN.

**Figure S2.**
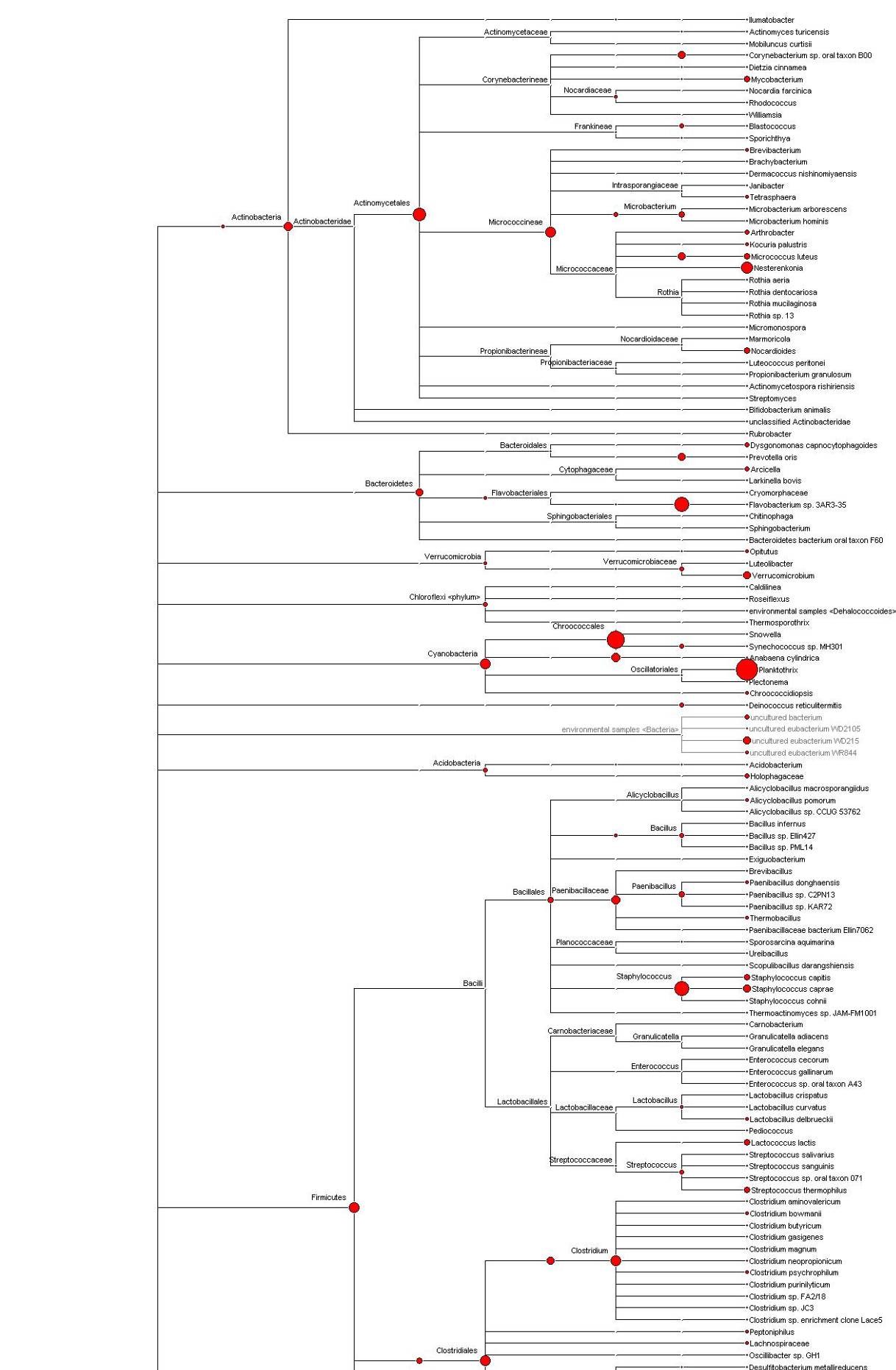


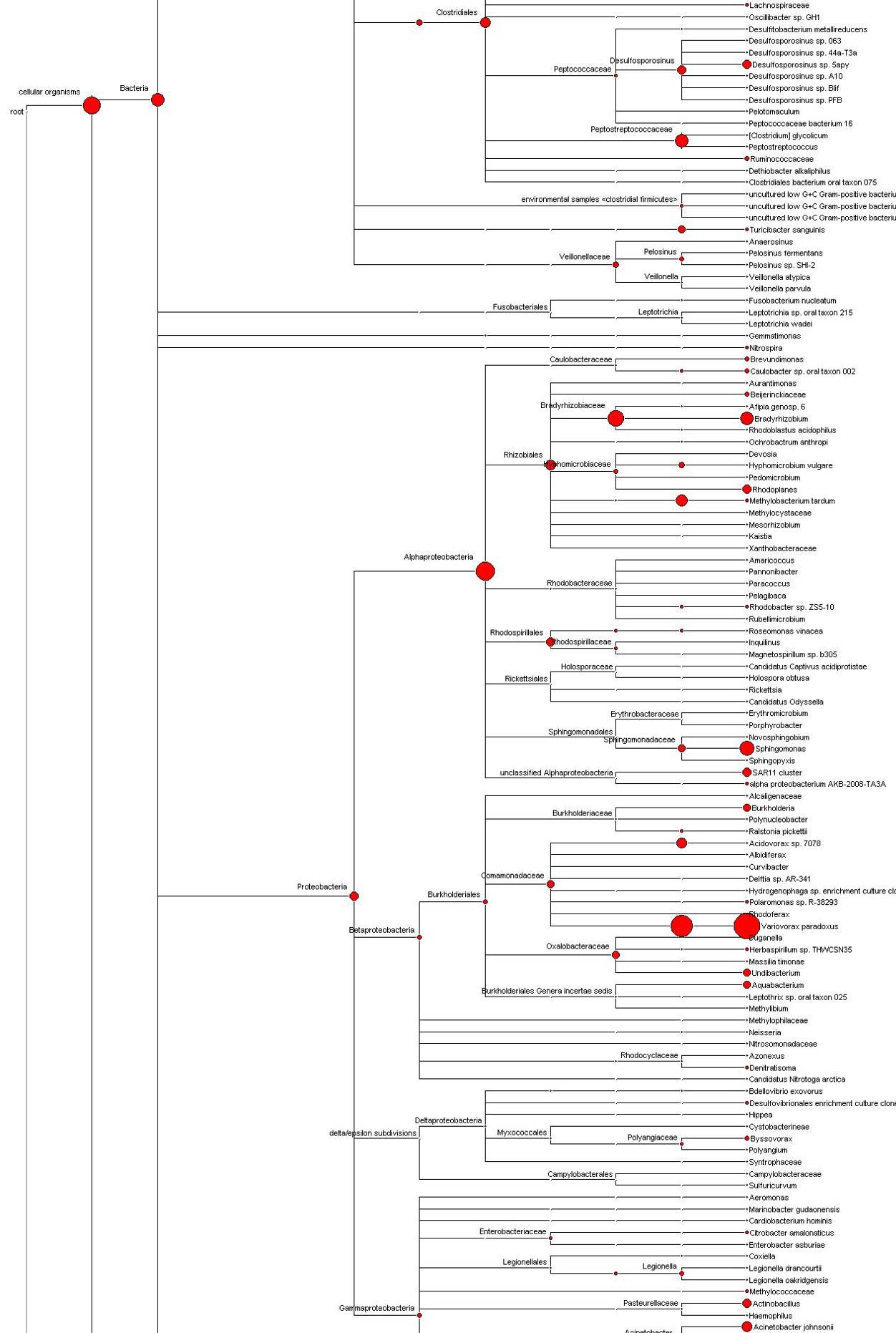


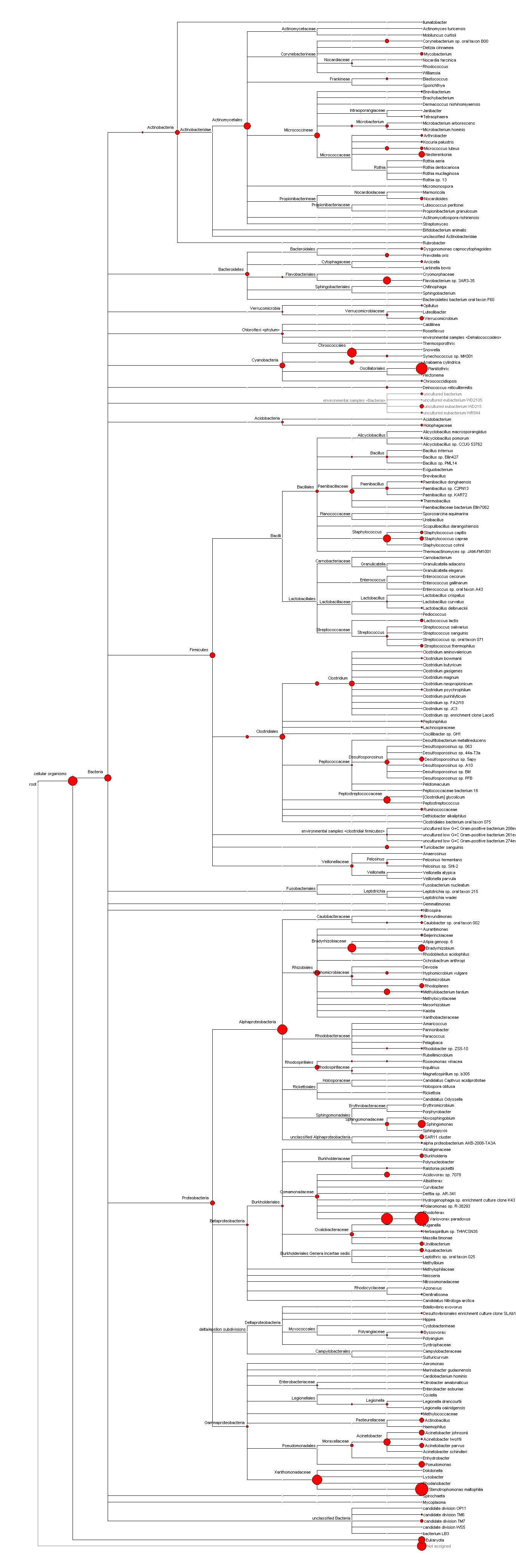

Supplement: Figure S2 — Overview of the 16S rRNA gene sequence set displayed by MEGAN. The species detected by the 454 high throughput sequencing of 16S rRNA high variable regions were displayed as a schematic phylogenetic tree using the software MEGAN. (DOC) [file pone.0106510.s002.doc]
