# Supplementary material for: Use of High Throughput Sequencing and Light Microscopy Show Contrasting Results in a Study of Phytoplankton Occurrence in a Freshwater Environment
Source: PLoS One. 2014 Aug 29;9(8):e106510. doi: 10.1371/journal.pone.0106510 (PMC4149573; doi:10.1371/journal.pone.0106510)

**Figure S3. Overview of the 18S rRNA gene sequence set displayed by MEGAN.** All the high quality reads generated by the 454 high throughput sequencing of 18S rRNA complicons were assigned to a taxonomy and displayed as a schematic phylogenetic tree using the software MEGAN.

**Figure S3.**
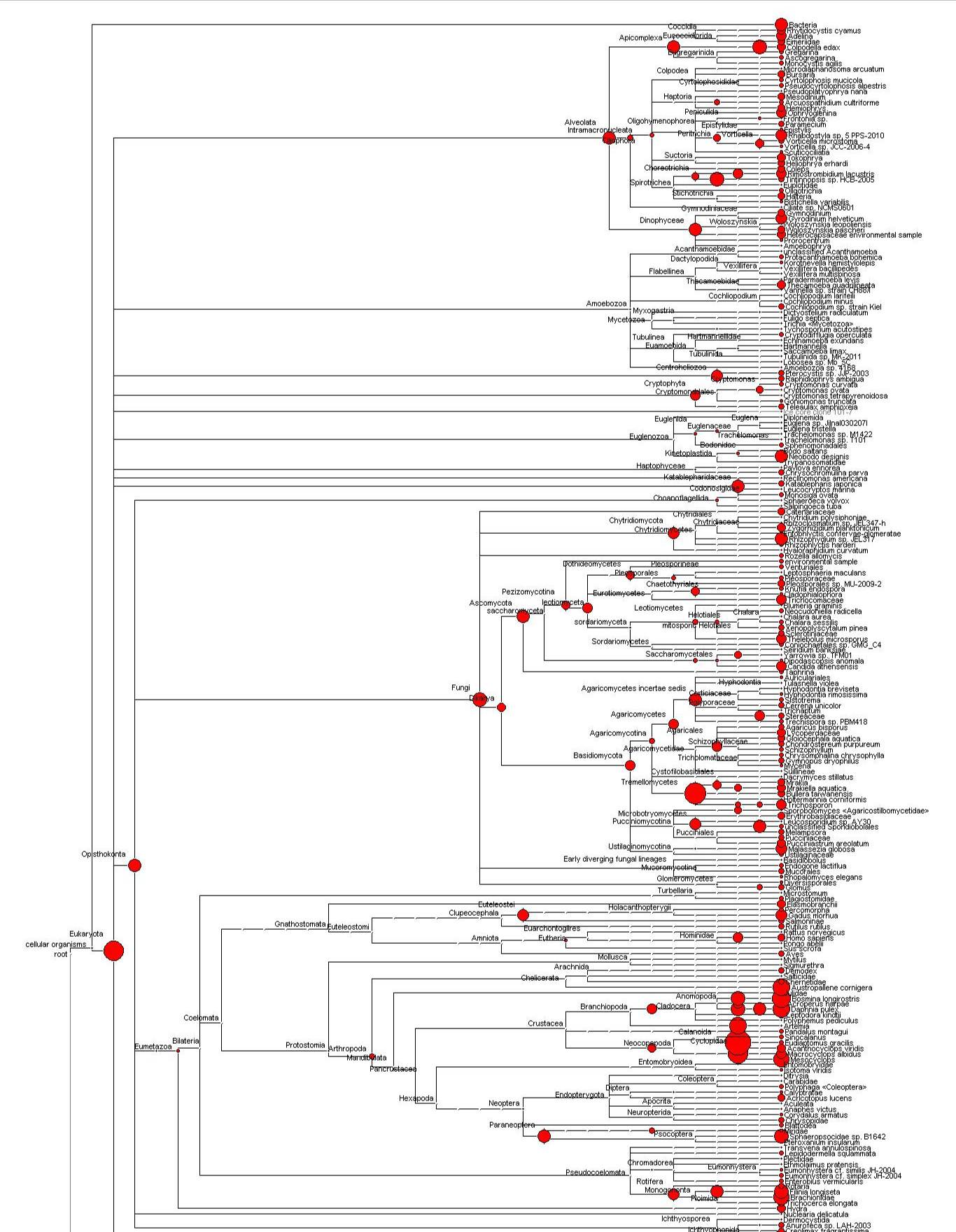


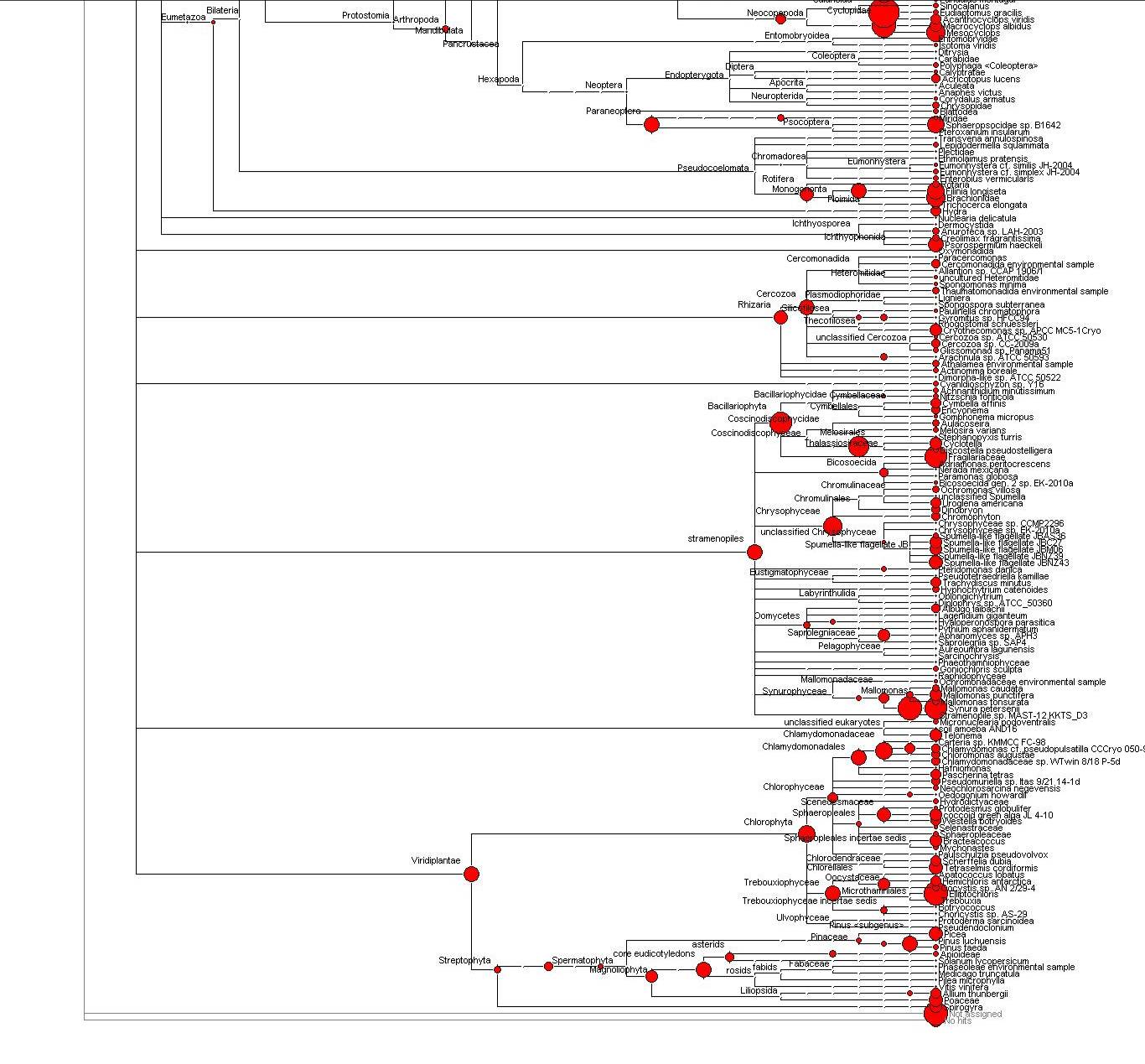

Supplement: Figure S3 — Overview of the 18S rRNA gene sequence set displayed by MEGAN. All the high quality reads generated by the 454 high throughput sequencing of 18S rRNA complicons were assigned to a taxonomy and displayed as a schematic phylogenetic tree using the software MEGAN. (DOC) [file pone.0106510.s003.doc]
