# Supplementary material for: Use of High Throughput Sequencing and Light Microscopy Show Contrasting Results in a Study of Phytoplankton Occurrence in a Freshwater Environment
Source: PLoS One. 2014 Aug 29;9(8):e106510. doi: 10.1371/journal.pone.0106510 (PMC4149573; doi:10.1371/journal.pone.0106510)

**Figure S4. The community structure of diatoms in our 18S rRNA gene sequence set (a) and the BLAST output against the nr database for reads assigned to “*Fragilariaceae*” (b) which gave many parallel results with the same scores and E-value.** It is possible that one representative sequence has two or more taxonomic best matches with an equal BLAST matching score. In that case this OTU could not get its final taxonomy assigned at a level below genus.

**Figure S4.**


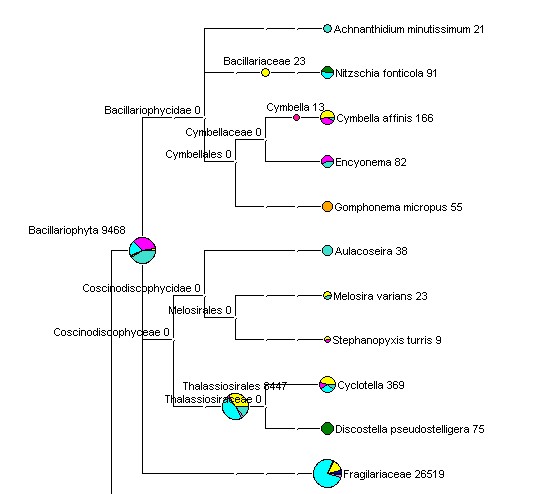


1. 18S sequence set

(b)


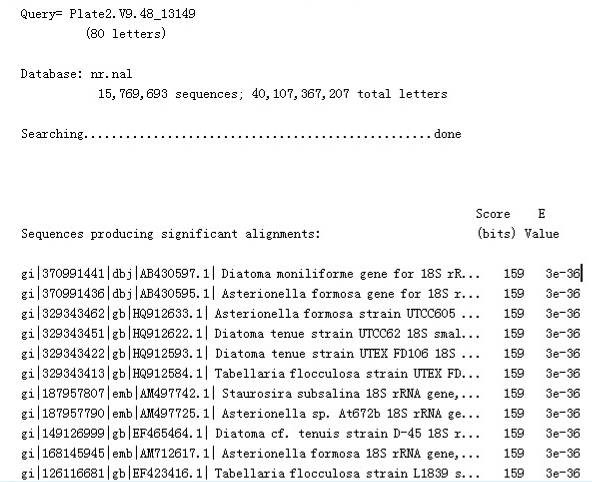

Supplement: Figure S4 — The community structure of diatoms in our 18S rRNA gene sequence set (a) and the BLAST output against the nr database for reads assigned to “ Fragilariaceae ” (b) which gave many parallel results with the same scores and E-value. It is possible that one representative sequence has two or more taxonomic best matches with an equal BLAST matching score. In that case this OTU could not get its final taxonomy assigned at a level below genus. (DOC) [file pone.0106510.s004.doc]
