# Supplementary material for: Use of High Throughput Sequencing and Light Microscopy Show Contrasting Results in a Study of Phytoplankton Occurrence in a Freshwater Environment
Source: PLoS One. 2014 Aug 29;9(8):e106510. doi: 10.1371/journal.pone.0106510 (PMC4149573; doi:10.1371/journal.pone.0106510)
Supplement: Table S1 — Sample descriptions of all the 300 filters used in sequencing approach. As described in the “Methods and Materials”, in total 300 different stored filters were used in our experiment, environmental DNAs extracted from these filters were pooled together as the DNA templates in the polymerase chain reactions before sequencing. The sampling date, year and sampling depth are all shown in this table. (DOC) [file pone.0106510.s005.doc]

**Table S1. Sample descriptions of all the 300 filters used in sequencing approach.** As described in the “Methods and Materials”, in total 300 different stored filters were used in our experiment, environmental DNAs extracted from these filters were pooled together as the DNA templates in the polymerase chain reactions before sequencing. The sampling date, year and sampling depth were all shown in this table.

| **Sample ID** | **Year** | **Date** | **Depth** | **Sample ID** | **Year** | **Date** | **Depth** | **Sample ID** | **Year** | **Date** | **Depth** | **Sample ID** | **Year** | **Date** | **Depth** |
| --- | --- | --- | --- | --- | --- | --- | --- | --- | --- | --- | --- | --- | --- | --- | --- |
| **G0001** | 1969 | 19-Mar-69 | 0 | **G0076** | 1973 | 19-Mar-73 | 1 | **G0151** | 1978 | 18-Sep-78 | 30 | **G0226** | 1983 | 21-Feb-83 | 35 |
| **G0002** | 1969 | 24-Mar-69 | 15 | **G0077** | 1973 | 19-Mar-73 | 15 | **G0152** | 1978 | 23-Oct-78 | 1 | **G0227** | 1983 | 21-Mar-83 | 1 |
| **G0003** | 1969 | 30-Apr-69 | 0 | **G0078** | 1973 | 23-Apr-73 | 1 | **G0153** | 1978 | 23-Oct-78 | 30 | **G0228** | 1983 | 21-Mar-83 | 6 |
| **G0004** | 1969 | 21-Apr-69 | 15 | **G0079** | 1973 | 23-Apr-73 | 15 | **G0154** | 1978 | 18-Oct-78 | 6 | **G0229** | 1983 | 21-Mar-83 | 35 |
| **G0005** | 1969 | 23-Apr-69 | 30 | **G0080** | 1973 | 21-May-73 | 1 | **G0155** | 1978 | 20-Nov-78 | 1 | **G0230** | 1983 | 18-Apr-83 | 1 |
| **G0006** | 1969 | 20-May-69 | 0 | **G0081** | 1973 | 21-May-73 | 15 | **G0156** | 1978 | 20-Nov-78 | 30 | **G0231** | 1983 | 18-Apr-83 | 6 |
| **G0007** | 1969 | 19-May-69 | 15 | **G0082** | 1973 | 21-May-73 | 30 | **G0157** | 1978 | 06-Dec-78 | 1 | **G0232** | 1983 | 18-Apr-83 | 35 |
| **G0008** | 1969 | 20-Jun-69 | 0 | **G0083** | 1973 | 18-Jun-73 | 1 | **G0158** | 1978 | 06-Dec-78 | 6 | **G0233** | 1983 | 24-May-83 | 1 |
| **G0009** | 1969 | 27-Jun-69 | 15 | **G0084** | 1973 | 18-Jun-73 | 15 | **G0159** | 1978 | 06-Dec-78 | 30 | **G0234** | 1983 | 24-May-83 | 6 |
| **G0010** | 1969 | 22-Jul-69 | 0 | **G0085** | 1973 | 18-Jun-73 | 30 | **G0160** | 1981 | 23-Jan-81 | 35 | **G0235** | 1983 | 24-May-83 | 35 |
| **G0011** | 1969 | 16-Jul-69 | 15 | **G0086** | 1973 | 16-Jul-73 | 1 | **G0161** | 1981 | 26-Jan-81 | 1 | **G0236** | 1983 | 20-Jun-83 | 1 |
| **G0012** | 1969 | 20-Aug-69 | 0 | **G0087** | 1973 | 16-Jul-73 | 15 | **G0162** | 1981 | 26-Jan-81 | 6 | **G0237** | 1983 | 20-Jun-83 | 6 |
| **G0013** | 1969 | 18-Aug-69 | 30 | **G0088** | 1973 | 16-Jul-73 | 30 | **G0163** | 1981 | 23-Feb-81 | 1 | **G0238** | 1983 | 20-Jun-83 | 35 |
| **G0014** | 1969 | 18-Sep-69 | 0 | **G0089** | 1973 | 20-Aug-73 | 1 | **G0164** | 1981 | 23-Feb-81 | 6 | **G0239** | 1983 | 18-Jul-83 | 1 |
| **G0015** | 1969 | 23-Sep-69 | 30 | **G0090** | 1973 | 20-Aug-73 | 30 | **G0165** | 1981 | 23-Mar-81 | 1 | **G0240** | 1983 | 18-Jul-83 | 6 |
| **G0016** | 1969 | 20-Oct-69 | 0 | **G0091** | 1973 | 24-Sep-73 | 1 | **G0166** | 1981 | 23-Mar-81 | 6 | **G0241** | 1983 | 18-Jul-83 | 35 |
| **G0017** | 1969 | 20-Oct-69 | 15 | **G0092** | 1973 | 24-Sep-73 | 15 | **G0167** | 1981 | 21-Apr-81 | 1 | **G0242** | 1983 | 22-Aug-83 | 1 |
| **G0018** | 1969 | 20-Nov-69 | 0 | **G0093** | 1973 | 24-Sep-73 | 30 | **G0168** | 1981 | 21-Apr-81 | 6 | **G0243** | 1983 | 22-Aug-83 | 6 |
| **G0019** | 1969 | 20-Dec-69 | 15 | **G0094** | 1973 | 22-Oct-73 | 1 | **G0169** | 1981 | 18-May-81 | 1 | **G0244** | 1983 | 22-Aug-83 | 36 |
| **G0020** | 1970 | 26-Jan-70 | 0 | **G0095** | 1973 | 22-Oct-73 | 15 | **G0170** | 1981 | 18-May-81 | 35 | **G0245** | 1983 | 19-Sep-83 | 1 |
| **G0021** | 1970 | 26-Jan-70 | 15 | **G0096** | 1973 | 22-Oct-73 | 30 | **G0171** | 1981 | 22-Jun-81 | 1 | **G0246** | 1983 | 19-Sep-83 | 6 |
| **G0022** | 1970 | 23-Feb-70 | 0 | **G0097** | 1973 | 26-Nov-73 | 1 | **G0172** | 1981 | 22-Jun-81 | 35 | **G0247** | 1983 | 19-Sep-83 | 36 |
| **G0023** | 1970 | 23-Feb-70 | 15 | **G0098** | 1973 | 26-Nov-73 | 15 | **G0173** | 1981 | 20-Jul-81 | 1 | **G0248** | 1983 | 24-Oct-83 | 1 |
| **G0024** | 1970 | 23-Mar-70 | 0 | **G0099** | 1973 | 26-Nov-73 | 30 | **G0174** | 1981 | 20-Jul-81 | 35 | **G0249** | 1983 | 24-Oct-83 | 6 |
| **G0025** | 1970 | 23-Mar-70 | 15 | **G0100** | 1973 | 17-Dec-73 | 1 | **G0175** | 1981 | 24-Aug-81 | 1 | **G0250** | 1983 | 24-Oct-83 | 36 |
| **G0026** | 1970 | 15-May-70 | 30 | **G0101** | 1973 | 17-Dec-73 | 15 | **G0176** | 1981 | 24-Aug-81 | 35 | **G0251** | 1983 | 21-Nov-83 | 1 |
| **G0027** | 1970 | 16-May-70 | 0 | **G0102** | 1976 | 16-Jan-76 | 6 | **G0177** | 1981 | 21-Sep-81 | 1 | **G0252** | 1983 | 21-Nov-83 | 6 |
| **G0028** | 1970 | 20-May-70 | 15 | **G0103** | 1976 | 19-Jan-76 | 1 | **G0178** | 1981 | 21-Sep-81 | 35 | **G0253** | 1983 | 21-Nov-83 | 36 |
| **G0029** | 1970 | 19-Jun-70 | 0 | **G0104** | 1976 | 19-Jan-76 | 30 | **G0179** | 1981 | 19-Oct-81 | 1 | **G0254** | 1983 | 19-Dec-83 | 1 |
| **G0030** | 1970 | 15-Jun-70 | 15 | **G0105** | 1976 | 23-Feb-76 | 1 | **G0180** | 1981 | 19-Oct-81 | 35 | **G0255** | 1983 | 19-Dec-83 | 6 |
| **G0031** | 1970 | 20-Jul-70 | 0 | **G0106** | 1976 | 23-Feb-76 | 30 | **G0181** | 1981 | 23-Nov-81 | 1 | **G0256** | 1983 | 19-Dec-83 | 36 |
| **G0032** | 1970 | 20-Jul-70 | 15 | **G0107** | 1976 | 27-Feb-76 | 6 | **G0182** | 1981 | 23-Nov-81 | 35 | **G0257** | 1989 | 23-Jan-89 | 1 |
| **G0033** | 1970 | 20-Aug-70 | 0 | **G0108** | 1976 | 22-Mar-76 | 1 | **G0183** | 1981 | 21-Dec-81 | 1 | **G0258** | 1989 | 23-Jan-89 | 6 |
| **G0034** | 1970 | 24-Aug-70 | 15 | **G0109** | 1976 | 22-Mar-76 |  | **G0184** | 1981 | 21-Dec-81 | 35 | **G0259** | 1989 | 23-Jan-89 | 36 |
| **G0035** | 1970 | 21-Sep-70 | 0 | **G0110** | 1976 | 20-Apr-76 | 1 | **G0185** | 1982 | 18-Jan-82 | 1 | **G0260** | 1989 | 20-Feb-89 | 1 |
| **G0036** | 1970 | 21-Sep-70 | 15 | **G0111** | 1976 | 20-Apr-76 | 6 | **G0186** | 1982 | 18-Jan-82 | 6 | **G0261** | 1989 | 20-Feb-89 | 6 |
| **G0037** | 1970 | 15-Oct-70 | 0 | **G0112** | 1976 | 18-May-76 | 1 | **G0187** | 1982 | 18-Jan-82 | 35 | **G0262** | 1989 | 20-Feb-89 | 36 |
| **G0038** | 1970 | 19-Oct-70 | 15 | **G0113** | 1976 | 18-May-76 | 6 | **G0188** | 1982 | 22-Feb-82 | 1 | **G0263** | 1989 | 20-Mar-89 | 1 |
| **G0039** | 1970 | 20-Oct-70 | 0 | **G0114** | 1976 | 19-May-76 | 6.3 | **G0189** | 1982 | 22-Feb-82 | 6 | **G0264** | 1989 | 20-Mar-89 | 6 |
| **G0040** | 1970 | 20-Oct-70 | 15 | **G0115** | 1976 | 21-Jun-76 | 1 | **G0190** | 1982 | 22-Feb-82 | 35 | **G0265** | 1989 | 20-Mar-89 | 36 |
| **G0041** | 1970 | 20-Nov-70 | 0 | **G0116** | 1976 | 21-Jun-76 |  | **G0191** | 1982 | 22-Mar-82 | 1 | **G0266** | 1989 | 24-Apr-89 | 1 |
| **G0042** | 1970 | 21-Nov-70 | 15 | **G0117** | 1976 | 19-Jul-76 | 1 | **G0192** | 1982 | 22-Mar-82 | 6 | **G0267** | 1989 | 24-Apr-89 | 6 |
| **G0043** | 1970 | 15-Dec-70 | 0 | **G0118** | 1976 | 19-Jul-76 | 30 | **G0193** | 1982 | 22-Mar-82 | 35 | **G0268** | 1989 | 24-Apr-89 | 36 |
| **G0044** | 1970 | 21-Dec-70 | 15 | **G0119** | 1976 | 23-Aug-76 | 1 | **G0194** | 1982 | 19-Apr-82 | 1 | **G0269** | 1989 | 22-May-89 | 1 |
| **G0045** | 1971 | 19-Jan-71 | 15 | **G0120** | 1976 | 23-Aug-76 | 30 | **G0195** | 1982 | 19-Apr-82 | 6 | **G0270** | 1989 | 22-May-89 | 6 |
| **G0046** | 1971 | 22-Feb-71 | 15 | **G0121** | 1976 | 20-Sep-76 | 1 | **G0196** | 1982 | 19-Apr-82 | 35 | **G0271** | 1989 | 22-May-89 | 36 |
| **G0047** | 1971 | 22-Mar-71 | 15 | **G0122** | 1976 | 20-Sep-76 | 6 | **G0197** | 1982 | 24-May-82 | 1 | **G0272** | 1989 | 19-Jun-89 | 1 |
| **G0048** | 1971 | 23-Mar-71 | 30 | **G0123** | 1976 | 20-Sep-76 | 30 | **G0198** | 1982 | 24-May-82 | 6 | **G0273** | 1989 | 19-Jun-89 | 6 |
| **G0049** | 1971 | 21-Apr-71 | 15 | **G0124** | 1976 | 25-Oct-76 | 1 | **G0199** | 1982 | 24-May-82 | 35 | **G0274** | 1989 | 19-Jun-89 | 36 |
| **G0050** | 1971 | 21-May-71 | 15 | **G0125** | 1976 | 25-Oct-76 | 6 | **G0200** | 1982 | 21-Jun-82 | 1 | **G0275** | 1989 | 24-Jul-89 | 1 |
| **G0051** | 1971 | 22-Jun-71 | 15 | **G0126** | 1976 | 25-Oct-76 | 30 | **G0201** | 1982 | 21-Jun-82 | 6 | **G0276** | 1989 | 24-Jul-89 | 6 |
| **G0052** | 1971 | 19-Jul-71 | 1 | **G0127** | 1976 | 22-Nov-76 | 1 | **G0202** | 1982 | 21-Jun-82 | 35 | **G0277** | 1989 | 24-Jul-89 | 36 |
| **G0053** | 1971 | 19-Jul-71 | 15 | **G0128** | 1976 | 22-Nov-76 | 30 | **G0203** | 1982 | 19-Jul-82 | 1 | **G0278** | 1989 | 21-Aug-89 | 1 |
| **G0054** | 1971 | 19-Jul-71 | 30 | **G0129** | 1976 | 27-Dec-76 | 1 | **G0204** | 1982 | 19-Jul-82 | 6 | **G0279** | 1989 | 21-Aug-89 | 6 |
| **G0055** | 1971 | 23-Aug-71 | 1 | **G0130** | 1976 | 27-Dec-76 | 6 | **G0205** | 1982 | 19-Jul-82 | 35 | **G0280** | 1989 | 21-Aug-89 | 36 |
| **G0056** | 1971 | 23-Aug-71 | 15 | **G0131** | 1976 | 27-Dec-76 | 30 | **G0206** | 1982 | 23-Aug-82 | 1 | **G0281** | 1989 | 18-Sep-89 | 1 |
| **G0057** | 1971 | 23-Aug-71 | 30 | **G0132** | 1978 | 23-Jan-78 | 1 | **G0207** | 1982 | 23-Aug-82 | 6 | **G0282** | 1989 | 18-Sep-89 | 6 |
| **G0058** | 1971 | 20-Sep-71 | 1 | **G0133** | 1978 | 23-Jan-78 | 30 | **G0208** | 1982 | 23-Aug-82 | 35 | **G0283** | 1989 | 18-Sep-89 | 36 |
| **G0059** | 1971 | 20-Sep-71 | 15 | **G0134** | 1978 | 20-Feb-78 | 1 | **G0209** | 1982 | 20-Sep-82 | 1 | **G0284** | 1989 | 23-Oct-89 | 1 |
| **G0060** | 1971 | 25-Oct-71 | 1 | **G0135** | 1978 | 20-Feb-78 | 6 | **G0210** | 1982 | 20-Sep-82 | 6 | **G0285** | 1989 | 23-Oct-89 | 6 |
| **G0061** | 1971 | 25-Oct-71 | 15 | **G0136** | 1978 | 20-Feb-78 | 30 | **G0211** | 1982 | 20-Sep-82 | 35 | **G0286** | 1989 | 23-Oct-89 | 36 |
| **G0062** | 1971 | 22-Nov-71 | 1 | **G0137** | 1978 | 20-Mar-78 | 1 | **G0212** | 1982 | 18-Oct-82 | 1 | **G0287** | 1989 | 20-Nov-89 | 1 |
| **G0063** | 1971 | 22-Nov-71 | 15 | **G0138** | 1978 | 20-Mar-78 | 30 | **G0213** | 1982 | 18-Oct-82 | 6 | **G0288** | 1989 | 20-Nov-89 | 6 |
| **G0064** | 1971 | 20-Dec-71 | 1 | **G0139** | 1978 | 24-Apr-78 | 1 | **G0214** | 1982 | 18-Oct-82 | 35 | **G0289** | 1989 | 20-Nov-89 | 36 |
| **G0065** | 1971 | 20-Dec-71 | 15 | **G0140** | 1978 | 24-Apr-78 | 30 | **G0215** | 1982 | 22-Nov-82 | 1 | **G0290** | 1989 | 18-Dec-89 | 1 |
| **G0066** | 1971 | 18-Jan-71 | 0 | **G0141** | 1978 | 22-May-78 | 1 | **G0216** | 1982 | 22-Nov-82 | 6 | **G0291** | 1989 | 18-Dec-89 | 6 |
| **G0067** | 1971 | 25-Feb-71 | 1 | **G0142** | 1978 | 22-May-78 | 30 | **G0217** | 1982 | 22-Nov-82 | 35 | **G0292** | 1989 | 18-Dec-89 | 36 |
| **G0068** | 1971 | 22-Mar-71 | 1 | **G0143** | 1978 | 19-Jun-78 | 1 | **G0218** | 1982 | 20-Dec-82 | 1 | **G0300** | 1970 | 29-Oct-70 | 15 |
| **G0069** | 1971 | 21-Apr-71 | 1 | **G0144** | 1978 | 19-Jun-78 | 30 | **G0219** | 1982 | 20-Dec-82 | 6 | **G0301** | 1970 | 22-Nov-70 | 15 |
| **G0070** | 1971 | 21-May-71 | 1 | **G0145** | 1978 | 24-Jul-78 | 1 | **G0220** | 1982 | 20-Dec-82 | 35 | **G0302** | 1970 | 23-Nov-70 | 15 |
| **G0071** | 1971 | 22-Jun-71 | 1 | **G0146** | 1978 | 24-Jul-78 | 30 | **G0221** | 1983 | 24-Jan-83 | 1 | **G0303** | 1970 | 26-Nov-70 | 15 |
| **G0072** | 1973 | 22-Jan-73 | 1 | **G0147** | 1978 | 10-Aug-78 | 6 | **G0222** | 1983 | 24-Jan-83 | 6 | **G0304** | 1970 | 26-Nov-70 | 0 |
| **G0073** | 1973 | 22-Jan-73 | 15 | **G0148** | 1978 | 21-Aug-78 | 1 | **G0223** | 1983 | 24-Jan-83 | 35 | **G0305** | 1970 | 27-Nov-70 | 15 |
| **G0074** | 1973 | 19-Feb-73 | 1 | **G0149** | 1978 | 21-Aug-78 | 30 | **G0224** | 1983 | 21-Feb-83 | 1 | **G0306** | 1970 | 04-Dec-70 | 15 |
| **G0075** | 1973 | 19-Feb-73 | 15 | **G0150** | 1978 | 18-Sep-78 | 1 | **G0225** | 1983 | 21-Feb-83 | 6 | **G0307** | 1970 | 07-Dec-70 | 15 |
