# Supplementary material for: Use of High Throughput Sequencing and Light Microscopy Show Contrasting Results in a Study of Phytoplankton Occurrence in a Freshwater Environment
Source: PLoS One. 2014 Aug 29;9(8):e106510. doi: 10.1371/journal.pone.0106510 (PMC4149573; doi:10.1371/journal.pone.0106510)
Supplement: Table S2 — Number of total and unique sequences during the 16S rRNA gene and 18S rRNA gene sequence processing. Several different steps such as denoising and chimera checking were carried out during sequence processing (see also Figure S1), low quality reads were filtered out by bioinformatics treatment, all the numbers of remaining sequences (and corresponding unique sequences) were recorded. (DOC) [file pone.0106510.s006.doc]

**Table S2. Number of total and unique sequences during the 16S and 18S sequence processing.** Several different steps such as denoising and chimera checking were carried out during sequence processing (see also Figure S1), low quality reads were filtered out by bioinformatics treatment, all the numbers of remaining sequences (and corresponding unique sequences) were recorded.

|  | Denoised output | | | | Screen+filtered output | | | | Precluster | | |  | Chimeras removed | | | | Clean Dataset | | |  |
| --- | --- | --- | --- | --- | --- | --- | --- | --- | --- | --- | --- | --- | --- | --- | --- | --- | --- | --- | --- | --- |
|  | Total |  | Unique |  | Total |  | Unique |  | Total |  | Unique |  | Total |  | Unique |  | Total |  | Unique |  |
| **16s** | 41998 |  | 2702 |  | 41751 |  | 2540 |  | 41751 |  | 2258 |  | -889 |  | -271 |  | 40862 |  | 1987 |  |
| **18s** | 180230 |  | 5191 |  | 145046 |  | 2707 |  | 145046 |  | 2245 |  | -11 |  | -5 |  | 145035 |  | 2240 |  |
