# Supplementary material for: Use of High Throughput Sequencing and Light Microscopy Show Contrasting Results in a Study of Phytoplankton Occurrence in a Freshwater Environment
Source: PLoS One. 2014 Aug 29;9(8):e106510. doi: 10.1371/journal.pone.0106510 (PMC4149573; doi:10.1371/journal.pone.0106510)
Supplement: Table S3 — Part of microscopy dataset. More than 5000 records were listed in the whole microscopy dataset. As shown in this sample table, each record includes the following information: (1) sampling date; (2) sampling depth; (3) species code (which could be translated to species name using a code - taxonomy table); (4) phytoplankton class; (5) bio-volume. (DOC) [file pone.0106510.s007.doc]

**Table S3. Part of microscopy dataset.** More than 5000 records were listed in the whole microscopy dataset. As shown in this sample table, each record includes the following information: (1) sampling date; (2) sampling depth; (3) species code (which could be translated to species name using a code - taxonomy table); (4) phytoplankton class; (5) bio-volume.

| **Sampling date** | **Depth** | **Species Code** | **Group** | **Bio-volume** |
| --- | --- | --- | --- | --- |
| 1969/6/25 | 0-10 | NITZ ACI | BACILLAR | 11.9 |
| 1969/6/25 | 0-10 | SYNE ACR | BACILLAR | 3.3 |
| 1969/6/25 | 0-10 | SYNE ACA | BACILLAR | 20.4 |
| 1969/6/25 | 0-10 | FRAG CRO | BACILLAR | 154.7 |
| 1969/6/25 | 0-10 | SYNE ULN | BACILLAR | 18.2 |
| 1969/6/25 | 0-10 | TABE FEN | BACILLAR | 944.7 |
| 1969/6/25 | 0-10 | SYNE ACU | BACILLAR | 5.8 |
| 1969/6/25 | 0-10 | SYNEDRA4 | BACILLAR | 45.4 |
| 1969/6/25 | 0-10 | EUDO ELE | CHLOROPH | 5.2 |
| 1969/6/25 | 0-10 | PAND MOR | CHLOROPH | 10.4 |
| 1969/6/25 | 0-10 | STAURASZ | CHLOROPH | 6.5 |
| 1969/6/25 | 0-10 | STAU PLA | CHLOROPH | 2.3 |
| 1969/6/25 | 0-10 | SCEN ARM | CHLOROPH | 3.4 |
| 1969/6/25 | 0-10 | SCENEDE1 | CHLOROPH | 3.6 |
| 1969/6/25 | 0-10 | SMACHRYS | CHRYSOPH | 0.9 |
| 1969/6/25 | 0-10 | STOCHRYS | CHRYSOPH | 2.0 |
| 1969/6/25 | 0-10 | ANAB CIR | CYANOPHY | 1004.3 |
| 1969/6/25 | 0-10 | APHA FLO | CYANOPHY | 28.3 |
| 1969/6/25 | 0-10 | OSCI AGA | CYANOPHY | 359.4 |
| 1969/6/25 | 0-10 | GOMP LAC | CYANOPHY | 9.1 |
| 1969/7/8 | 0-10 | SYNE ACR | BACILLAR | 4.5 |
| 1969/7/8 | 0-10 | FRAG CRO | BACILLAR | 214.9 |
| 1969/7/8 | 0-10 | SYNE ACU | BACILLAR | 7.4 |
| 1969/7/8 | 0-10 | PAND MOR | CHLOROPH | 28.6 |
| 1969/7/8 | 0-10 | STAURASZ | CHLOROPH | 26.1 |
| 1969/7/8 | 0-10 | EUDO ELE | CHLOROPH | 15.6 |
| 1969/7/8 | 0-10 | SCEN ARM | CHLOROPH | 1.1 |
| 1969/7/8 | 0-10 | STAU PLA | CHLOROPH | 25.4 |
| 1969/7/8 | 0-10 | STOCHRYS | CHRYSOPH | 0.6 |
| 1969/7/8 | 0-10 | APHA FLO | CYANOPHY | 19.6 |
| 1969/7/8 | 0-10 | OSCI AGA | CYANOPHY | 544.5 |
| 1969/7/8 | 0-10 | ANAB CIR | CYANOPHY | 69.6 |
| 1969/7/8 | 0-10 | GOMP LAC | CYANOPHY | 751.4 |
| 1969/7/14 | 0-10 | FRAG CRO | BACILLAR | 132.8 |
| 1969/7/14 | 0-10 | SYNE ACA | BACILLAR | 2.3 |
| 1969/7/14 | 0-10 | STAURASZ | CHLOROPH | 13.1 |
| … | … | … | … | … |
| (1969 – 1990) | … | … | … | … |
| … | … | … | … | … |
| 1990/10/18 | 0-10 | RHOD LEN | CRYPTOPH | 3.7 |
| 1990/10/18 | 0-10 | APHA FLO | CYANOPHY | 7.9 |
| 1990/10/18 | 0-10 | CHOO LIM | CYANOPHY | 37.8 |
| 1990/10/18 | 0-10 | ACHROONZ | CYANOPHY | 1.0 |
| 1990/10/18 | 0-10 | GYMN LAC | DINOPHYC | 2.8 |
| 1990/10/18 | 0-10 | GYMN HEL | DINOPHYC | 19.3 |
